# Theranostics: radionuclide imaging and therapy in neuroendocrine tumours

**DOI:** 10.1186/1470-7330-15-S1-O16

**Published:** 2015-10-02

**Authors:** Brent Drake, Thomas Grüning

**Affiliations:** 1 Department of Nuclear Medicine, Plymouth Hospitals NHS Trust, Plymouth, Devon PL6 8DH, UK

## 

Neuroendocrine tumours (NETs) are a heterogeneous group with significant variation in morphological characteristics and functional behaviour. This poses challenges in terms of both biochemical and imaging assessment.

Somatostatin receptor (sst) overexpression is documented in several malignancies. The sst subtype 2 (sst_2_) is overexpressed in NETs [1] and can be targeted for both somatostatin receptor scintigraphy and therapy.

In-111-octreotide (Octreoscan), a gamma imaging sst agent, binds with relatively high affinity to sst _2_.This has good overall sensitivity in the detection of NETs (80-100%) [2]. However, sensitivity varies according to tumour type, grade, and location (e.g. sensitivity 24% in insulinomas) [3].

PET agents such as Ga-68-DOTA-labelled somatostatin analogues have been developed with higher receptor affinity when compared to gamma-based agents. This leads to improved target-to-background ratio and provides the improved imaging characteristics inherent of PET tracers. A 2012 meta-analysis demonstrated pooled sensitivity and specificity in detecting NETs of 93% and 91%, respectively [4] with higher detection rates compared to conventional sst imaging [5].

Whilst other PET tracers such as F-18-DOPA have shown utility and higher sensitivity than conventional sst scintigraphy, their role is limited to problem solving at present. F-18-FDG can, however, provide prognostic information with patients showing uptake having a progression free survival of 0% at 2 years compared with 75±10% in those without uptake [6].

Peptide receptor radionuclide therapy (PRRT) utilises primarily beta-emitting radioisotopes such as Lu-177 and Y-90 linked to somatostatin analogues such as DOTATATE and DOTATOC. Whilst phase 3 data comparing PRRT with other therapies is awaited, treatment with Y-90 and Lu-177 PRRT has been shown to have response rates of approximately 80% and to confer a survival advantage over historical controls [7]. Unsurprisingly, extensive hepatic involvement is an adverse factor for progression free survival, whereas high tumour uptake in pre-therapy imaging confers prolonged survival [8].

Due to the differences in beta particle energy and path length, it has been postulated that Lu-177 PRRT would be best suited to smaller tumour volumes compared with Y-90 which emits a more energetic particle with longer path length. Kunikowska and colleagues have now demonstrated that overall survival is significantly higher in patients treated with combination Y-90/Lu-177-DOTATATE compared with Y-90-DOTATATE alone [9].

Research into the role of sst antagonists is relatively new. Several studies have shown a significantly greater number of binding sites for antagonists when compared to agonists such as Lu-177-DOTATATE. Pilot studies have shown 1.7-10.6 times higher tumour dose with the antagonist Lu-177-DOTA-JR11 when compared to Lu-177-DOTATATE [10]. Encouraging results have also been obtained with the use of radiosensitising chemotherapeutic agents administered together with PRRT. A phase 2 study evaluating capecitabine with Lu-177-octreotate demonstrated a response rate of 94% with no significant increase in toxicity [11]. Further work is required, however, these advances are likely to play a significant role in the future.
